# Network Pharmacology and Machine Learning Identify Flavonoids as Potential Senotherapeutics

**DOI:** 10.3390/ph18081176

**Published:** 2025-08-09

**Authors:** Jose Alberto Santiago-de-la-Cruz, Nadia Alejandra Rivero-Segura, María Elizbeth Alvarez-Sánchez, Juan Carlos Gomez-Verjan

**Affiliations:** 1 Dirección de Investigación, Instituto Nacional Geriatría (INGER), Mexico City 10200, Mexico; alberto.santiago@alumnos.uacm.edu.mx (J.A.S.-d.-l.-C.); nrivero@inger.gob.mx (N.A.R.-S.); 2 Posgrado en Ciencias Genómicas, Universidad Autónoma de la Ciudad de México, San Lorenzo 290, Col. Del Valle, Mexico City 03100, Mexico; maria.alvarez@uacm.edu.mx

**Keywords:** senescence, flavonoids, network pharmacology, machine learning, ageing

## Abstract

**Background/Objectives:** Cellular senescence is characterised by irreversible cell cycle arrest and the secretion of a proinflammatory phenotype. In recent years, senescent cell accumulation and senescence-associated secretory phenotype (SASP) secretion have been linked to the onset of chronic degenerative diseases associated with ageing. In this context, the senotherapeutic compounds have emerged as promising drugs that specifically eliminate senescent cells (senolytics) or diminish the damage caused by SASP (senomorphics). On the other hand, computational approaches, such as network pharmacology and machine learning, have revolutionised the identification of novel drugs. These tools enable the analysis of large volumes of compounds and the optimisation of the search for the most promising ones as potential drugs. Therefore, we employed such approaches in the present study to identify potential senotherapeutic compounds. **Methods:** First, we constructed drug-protein interaction networks related to cellular senescence. Then, using three machine learning models (Random Forest, Support Vector Machine, and K-Nearest Neighbours), we classified these compounds based on their therapeutic potential against senescence. **Results:** Our results enabled us to identify 714 compounds with potential senescent therapeutic activity, of which 270 exhibited desirable medicinal chemistry properties, and we developed an interactive web tool freely accessible to the scientific community. **Conclusions:** we found that flavonoids were the most abundant compound class from which 18 have never been reported as senotherapeutics.

## 1. Introduction

Cellular senescence is an irreversible state of proliferative arrest characterised by various morphological, biochemical, and genetic changes [1]. In this sense, although senescence initially contributes to developmental processes of tissue repair, regeneration, and immune surveillance, it also induces detrimental effects in the organism, contributing to the development of the most common age-related diseases. Moreover, senescent cells secrete numerous factors, collectively referred to as the senescence-associated secretory phenotype (SASP), which includes proinflammatory cytokines, chemokines, proteases, and inhibitory molecules [2]. The molecular mechanisms leading to cellular senescence are diverse and complex, encompassing DNA damage, telomeric shortening, oncogene activation, mutation accumulation, increased reactive oxygen species (ROS), and mitochondrial dysfunction [3].

The dysfunction in senescent cells (SCs) elimination intensifies with age, leading to their tissue accumulation and increased senescence-associated secretory phenotype (SASP), which perpetuates molecular damage in a feedback loop that accelerates ageing [4,5]. Concomitantly, immunosenescence, the decline of the immune system in number and function, reduces the number and activity of immune cells, weakening the recognition and elimination of SCs through NK cells, macrophages, and cytotoxic T lymphocytes [6]. Simultaneously, accumulated SCs secrete pro-inflammatory cytokines, chemokines, and growth factors that contribute to the chronic low-grade inflammatory state (inflammaging), further deteriorating immune function and creating a vicious cycle between cellular senescence and immunosenescence [7]. They are also linked to neurodegenerative and cardiovascular diseases, as well as some types of cancer [8]. This bidirectional interaction not only accelerates the ageing process but also increases susceptibility to age-related diseases, underscoring the critical importance of developing geroprotective therapies that can disrupt these interconnected pathological mechanisms [9].

In recent years, senotherapy has emerged as a promising pharmacological strategy for healthy ageing [9,10], focusing on two main strategies: senolytics, which eliminate senescent cells, and senomorphics, which inhibit the adverse effects of SASP [11,12]. Among the most studied senotherapeutics are metformin [13], navitoclax [14], quercetin, and dasatinib [15]. However, only a combination of dasatinib and quercetin has reached clinical trials [16,17].

The development of senotherapeutic drugs faces significant challenges due to the specificity and potential toxicity in non-senescent cells [18]. To address these limitations, current research is focusing on two innovative approaches: network pharmacology [19] and machine learning (ML) [20]. Network pharmacology is an approach that uses systems biology to identify and predict multiple drug targets in complex treatments, focusing on interactions within signalling networks [20]. In contrast, machine learning is a fundamental branch of artificial intelligence, which seeks to develop algorithms and models that enable computer systems to learn from data, optimising their performance, such as category classification or regression [21]. Interestingly, in recent years, both network pharmacology and machine learning have been applied for target identification, drug discovery and optimisation, predicting drug-target interactions, and virtual screening of vast libraries of chemical compounds to identify promising candidates before they are synthesised and tested [22,23,24,25]. In this context, previous studies reported the use of chemoinformatics analysis based on chemical descriptors and pharmacokinetic properties to identify a set of natural compounds as candidates against cellular senescence [9]; nevertheless, many of the targets of these identified drugs have not yet been fully explored, meaning that other putative senolytics identified through fingerprint analysis, but not selected in this study, might also exhibit senolytic activity due to their structural similarity. Besides, they only restrict the use of network pharmacology for the identification of potential senolytic candidates, increasing the bias in the results. Conversely, Smer-Barreto et al. employed machine learning techniques to identify three senolytics—ginkgetin, oleandrin, and periplocin—which were validated in vitro [20]. However, the study notes that oleandrin is cardiotoxic and that further studies are required to ensure the safety of such compounds. This limitation arises from the fact that the authors focused solely on using machine learning algorithms to identify potential senolytic compounds, disregarding relevant pharmacological properties. Another limitation of the study is the class imbalance in the training data, with non-senolytics (negative) overrepresented relative to senolytics (positive), which leads to inflated performance metrics. Overall, this evidence suggests that using both tools together could be a novel strategy for accelerating drug discovery and developing new therapies that could improve quality of life during ageing by reducing the number of experiments required and enhancing the types of molecules screened. Therefore, the present study aims to identify chemical compounds with potential senotherapeutic activity by combining network pharmacology and supervised machine learning approaches.

## 2. Results

**Figure 1:**
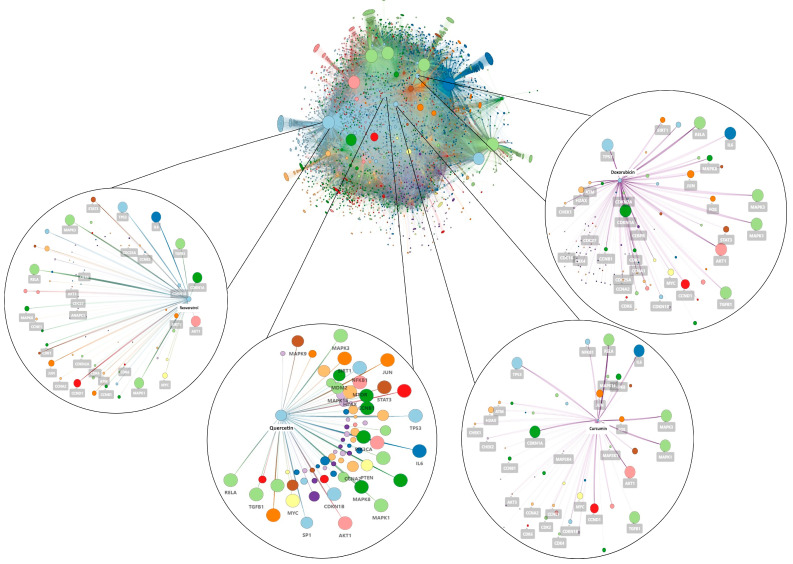
Drug-protein senescence interactome. The interactome shows the connections between the chemical compounds (65,339) and the 249 proteins associated with senescence. Nodes represent proteins and chemicals. The colour of the nodes is random. The edges indicate the different interactions between chemicals and proteins as reported by CTD without any size. The size of the nodes is approximately proportional to their degree value.

We mapped 249 proteins involved in cellular senescence that were imported into CTD to obtain gene-compound interactions. Once we eliminated redundancy and non-human species, we found that 65,339 compounds interact with proteins related to cellular senescence (Figure 1). The interactome represents proteins or chemical compounds as nodes, while the edges represent the interactions between these nodes, with all edges having the same weight. The largest nodes are key proteins or compounds that act as hubs within the interaction network, including p53, ERK2, ERK1, TGF-β1, IL-6, AKT1, mTOR, cyclin D1, and p21; additionally, Resveratrol, Quercetin, Curcumin, Doxorubicin, Cisplatin, and Azoles were also detected.

Subsequently, we identify the SMILES of 54,401 compounds out of 65,339 total compounds, for which 39 molecular descriptors were calculated, as described in the methods section. The RF, SVM, and KNN models were evaluated using the metrics accuracy, specificity, precision, Recall, F1-score, and Kappa value (Table 1).

Our results indicate that the RF model obtained the best overall performance among the three algorithms evaluated. It received an accuracy of 0.88, suggesting that it correctly classified 88% of the cases. It achieved a specificity of 0.92, indicating a high ability to identify negatives correctly. Additionally, the Kappa coefficient for this model was 0.76, confirming substantial agreement and suggesting that the model performs significantly better than chance.

**Figure 2:**
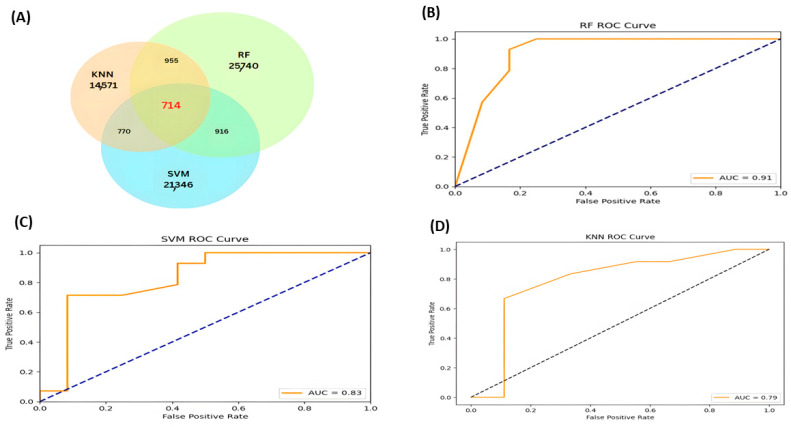
Venn diagram and ROC curves of the ML models. (**A**) Venn diagram of the compounds classified across the three models as possible senotherapeutic molecules. (**B**) ROC curve of the RF model. (**C**) ROC curve of the SVM model. (**D**) ROC curve of the KNN model.

On the other hand, SVM and KNN demonstrated similar overall performance, with identical F1 scores (0.76), but distinct strengths. The SVM showed good sensitivity (0.83) but inferior accuracy and specificity, with a Kappa of 0.54 indicating moderate agreement. KNN was notable for its accuracy (0.88) and specificity (0.88), but had a low sensitivity (0.67), making it more suitable for minimising false positives. Therefore, to enhance the robustness and accuracy of our classification, we selected only compounds classified as senotherapeutics by all three models (Figure 2).

Our results suggest that only 714 compounds were positively scored by the three ML models (Figure 2A). ROC curves illustrate the relationship between the actual and false favourable rates as the decision threshold of the model varies. The area under the curve (AUC) was evaluated, which indicates the probability that the ML model correctly classifies a randomly selected compound as positive; the closer this value is to 1, the better the model’s performance. Our AUC values are 0.91 for RF (Figure 2B), 0.83 for SVM (Figure 2C), and 0.79 for KNN (Figure 2D), indicating that our models effectively distinguish between categorical classes or classifications.

Once we filtered the compounds according to Lipinski’s rules, 270 compounds met the desired physicochemical characteristics. These compounds were stored in a freely accessible database published online on March 2025 (https://gcoixc-laboratorio0de0bioinform0tic-inger.shinyapps.io/Senotherapeutics_Shiny/).

**Figure 3:**
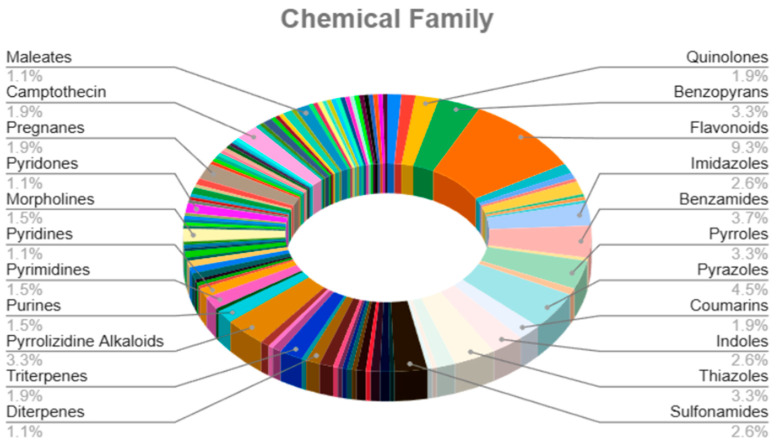
Compound family analysis of senotherapeutic compounds identified by ML. The pie chart shows the chemical families to which our senotherapeutics belong, highlighting flavonoids with the highest percentage.

Finally, we analysed the chemical family of these 270 compounds (Figure 3). Interestingly, 9.3% of the entire database consisted of flavonoids, from which only 18 flavonoids have not been previously reported as senotherapeutics (3′,4′,7-trihydroxyisoflavone, daidzin, catechin, eriodictyol, auriculasin, pomiferin, 4′-O-methylalpinumisoflavone, tephrosin, 5,7-dihydroxy-3-(3-hydroxy-4-methoxybenzyl)-6-methoxychroman-4-one, calycosin-7-O-beta-D-glucoside, (R)-hesperetin, glycitein, glycitin, jaceosidin, silybin, isosilybin A, eupafolin, and skullcapflavone II).

Finally, to validate the potential senolytic effect of flavonoids, the most abundant chemical family, as indicated by our results above, we performed a docking analysis. All proteins were selected since they have already been reported to interact with flavonoids. In this context, according to the results (Table 2 and Supplementary Materials) we obtain 18 blind couplings between the selected proteins (p53, c-Fos, Cyclin D1, Trx, p21, AKT1, CDK1, NORE1, p65, c-Jun, and p38α) and compounds (3′,4′,7-trihydroxyisoflavone, daidzin, catechin, eriodictyol, auriculasin, pomiferin, 4′-O-methylalpinumisoflavone, tephrosin, 5,7,3′-trihydroxy-3,4′-dimethoxyflavone, calycosin-7-O-beta-D-glucoside, 5,7-dihydroxy-3-(3-hydroxy-4-methoxybenzyl)-6-methoxychroman-4-one, glycitein, glycitin, jaceosidin, silybin, isosilybin A, eupafolin, and skullcapflavone II). Interestingly, their interactions exhibit a moderate affinity (values between −6 and −9 kcal/mol) [26], and only a few compounds show high affinity (tephrosin, 5,7-dihydroxy-3-(3-hydroxy-4-methoxybenzyl)-6-methoxychroman-4-one, and isosilybin A, as they exhibit values between −9 and −12 kcal/mol) or weak affinity (jaceosidin and eupafolin, which show values between −4 and −6 kcal/mol), suggesting that such compounds with high or moderate affinity may be considered for further experimental validation.

## 3. Discussion

Computational assays have revolutionised the pharmaceutical industry by offering innovative tools to speed up and optimise traditionally slow and costly processes. They do not replace traditional methods, but they are critical accelerators in the era of precision medicine. Their ability to analyse big data makes them pillars of modern pharmaceutical discovery.

In this sense, our research group employed a chemoinformatics analysis based on chemical descriptors and filtered by medicinal chemistry properties, such as Lipinski’s rules, to identify terpenoids and naphthalenes as natural compounds that could be candidates for combating cellular senescence [6]. On the other hand, using a machine learning approach based on a structure-activity relationship network, Smer-Barreto et al., 2023, identified three compounds with potential as senolytics (ginkgetin, oleandrin, and periplocin); interestingly, ginkgetin is a biflavonoid [20]. Such a study demonstrates the potential for integrating network pharmacology and machine learning (ML) to accelerate the discovery of senotherapeutics, a critical challenge in ageing medicine.

In this sense, our results demonstrate that the construction of the drug-protein interactome enabled the mapping of key molecular dynamics associated with senescence, highlighting central nodes such as p53, ERK2, TGF-β1, IL-6, and AKT1, which are essential in cell cycle regulation and the DNA damage response [3]. These findings align with previous studies that have linked these proteins to SASP and their role in chronic diseases [1,2]. Moreover, it is essential to highlight that the compounds mentioned above are currently being investigated as potential senotherapeutics [27,28] or geroprotectors [29].

Our study identifies 270 compounds as candidates for use as senotherapeutic agents through the use of three machine learning models (RFC, SVM, and KNN), significantly reducing the number of molecules (65,339 compounds) to be experimentally analysed. Interestingly, 49 of these compounds are already FDA-approved drugs, opening up the possibility of their pharmacological repositioning. This strategy can effectively accelerate their validation as senotherapeutic agents. Moreover, the presence of antioxidants (e.g., resveratrol) and chemotherapeutics (e.g., fluorouracil) in the results reinforces the hypothesis that molecules with dual mechanisms of action (proapoptotic and antioxidant) could selectively modulate senescence, as already reported in preclinical studies by Hickson et al., who treated 15 patients with diabetic kidney disease with a combination of Dasatinib + Quercetine, most of whom had reduced numbers of senescent cells in blood and adipose tissue after treatment [16]. We developed a free web tool to disseminate the database of the 270 compounds that may be potential senolytic compounds. This database serves as a valuable resource for the scientific community, facilitating open access to data and fostering interdisciplinary collaborations.

Additionally, our study reveals that 9.3% of the compounds identified by ML models are flavonoids from which only 18 have not been previously reported as senotherapeutics (3′,4′,7-trihydroxyisoflavone, daidzin, catechin, eriodictyol, auriculasin, pomiferin, 4′-O-methylalpinumisoflavone, tephrosin, 5,7-dihydroxy-3-(3-hydroxy-4-methoxybenzyl)-6-methoxychroman-4-one, calycosin-7-O-beta-D-glucoside, (R)-hesperetin, glycitein, glycitin, jaceosidin, silybin, isosilybin A, eupafolin and skullcapflavone II). Such results are quite relevant since flavonoids exhibit a wide range of biological activities, including anticancer, anti-inflammatory, antidiabetic, and neuroprotective effects [30], suggesting that combining these compounds with other therapies may yield more effective senotherapies. Nevertheless, it is essential to mention that many flavonoids exhibit poor bioavailability due to their low aqueous solubility, poor absorption, metabolic transformation, and limited distribution in organs and tissues [31]. This suggests that if flavonoids are to be used as senotherapeutic agents, they need to be improved by structural transformations, absorption enhancers, or using pharmacological technologies as mentioned in [32] to improve pharmacokinetic properties. For instance, synthetic flavonoids have been recently developed to enhance the stability and modulation of platelet function [33].

Interestingly, upon performing the docking validation, we found that only tephrosin, 5,7-dihydroxy-3-(3-hydroxy-4-methoxybenzyl)-6-methoxychroman-4-one, and isosilybin A exhibit higher energetic values (−9 and −12 kcal/mol) between their receptors and the molecules. This suggests that these compounds may be suitable for further experimental analysis.

Finally, our results from the docking assay reveals that the identified flavonoid showed moderate to high binding affinity to senescence-related target proteins, many of which have been classified as PAINS (pan-assay interference compounds) or IMPS (invalid metabolic panaceas) [34,35], suggesting possible nonspecific effects in biological assays; flavonoids represent a remarkable template for the development of numerous compounds with diverse biological properties [36].

### Limitations of the Study

This study is based exclusively on computational analysis and therefore lacks experimental validation. Furthermore, some of the selected compounds, such as several flavonoids, have been reported in the literature as frequent interferents in biological assays (PAINS), with low bioavailability and unfavourable metabolism. Therefore, the findings presented should be considered exploratory and require additional experimental validation to confirm their senotherapeutic relevance.

## 4. Materials and Methods

### 4.1. Network Pharmacology

A drug-protein interactome was constructed to investigate the interactions between compounds and proteins involved in cellular senescence. To build this interactome, we identified 249 proteins relevant to cellular senescence using the freely accessed databases KEGG (https://www.genome.jp/kegg/pathway.html) (accessed on 11 November 2024) and Reactome (https://reactome.org/content/detail/R-HSA-2559583) (accessed on 11 November 2024), and duplicates were manually removed. We prioritise the use of KEGG and Reactome databases since both are constantly curated and updated, making them quite suitable for different research needs. To gain insight into the compounds interacting with these proteins, data on these interactions were extracted from the freely accessed database Comparative Toxicogenomics Database (CTD) (https://ctdbase.org) [37] (accessed on 11 November 2024), with a focus on interactions in humans. The network was constructed and analysed using Graphistry v1.0 (https://www.graphistry.com/) via the Application Programming Interface (API) in Python v3.0.

### 4.2. Molecular Descriptors

Molecular descriptors (1–3D) were calculated for each dataset using the freely available DataWarrior v5.5.0 software (https://openmolecules.org/datawarrior). We calculated the following physicochemical, medicinal chemistry, and toxicoinformatic properties [38]: Total Molweight, Monoisotopic Mass, cLogP, cLogS, H-Acceptors, H-Donors, Total Surface Area, Relative PSA, Polar Surface Area, Druglikeness, Shape Index, Molecular Flexibility, Molecular Complexity, Fragments, Non-H Atoms, Non-C/H Atoms, Metal-Atoms, Electronegative Atoms, Stereo Centers, Rotatable Bonds, Rings Closures, Aromatic Atoms, sp3-Atoms, Symmetric atoms, Small Rings, Carbo-Rings, Hetero-Rings, Saturated Rings, Non-Aromatic Rings, Aromatic Rings, Saturated Carbo-Rings, Non-Aromatic Carbo-Rings, Carbo-Aromatic Rings, Saturated Hetero-Rings, Non-Aromatic Hetero-Rings, Hetero-Aromatic Rings, Amides, Amines, Alkyl-Amines, Aromatic Amines, Aromatic Nitrogens, Basic Nitrogens, and Acidic Oxygens.

### 4.3. Supervised ML Analysis (SVM, KNN, and RFC)

Among ML algorithms, we found Random Forest (RF), which builds multiple decision trees during the training process and determines the most frequent class for classification [39]; Support Vector Machine (SVM), which searches for the optimal hyperplane to separate different classes of data in a high-dimensional space [40]; and K-Nearest Neighbors (KNN), which classifies data according to the predominant class of its nearest neighbours in the feature space [41].

We utilised the Scikit-learn package v1.7.1 (https://scikit-learn.org/stable/index.html) in Python 3.0 to generate the models, which implement various statistical and machine learning algorithms. We constructed a classifier using the training set data of already reported senotherapeutic compounds by Lei Zhang [42]. Additionally, to increase the specificity of our model, we used a set of non-related senotherapeutic compounds randomly extracted from the ChEMBL database (https://www.ebi.ac.uk/chembl/) accessed on 25 November 2024.

Subsequently, the data were divided into training (75%) and test (25%) subsets by stratified random sampling, with the subsets further divided into positive and negative senotherapeutics. We used three machine learning models (RF, SVM, and KNN) to generate three classifiers.

The receiver operating characteristic area under the curve (ROC-AUC) was used to evaluate the predictive accuracy of our models. We used a metric indicating the performance of the models, with values ranging from 0 to 1, where 1 represents perfect prediction. For each model’s accuracy, we calculated and assessed the overall effectiveness and specificity to determine whether true negatives were correctly classified. Similarly, we evaluated the balance between accuracy and sensitivity using the F1-score [43]. Additionally, to provide a more robust and nuanced evaluation of classification model performance, we calculated the Kappa statistics for each model.

Once we had the classifier for each algorithm, we used it to select the potential senotherapeutic compounds from the interactome obtained by network pharmacology, extracted from the CTD. We selected only those compounds that were shared across all three classifiers to enhance the robustness and accuracy of our calculations.

### 4.4. Druglikeness by Lipinski Rules

The compounds positively predicted by the three models were integrated and filtered using druglikeness analysis based on Lipinski’s rules [44] to preliminarily filter only those compounds with suitable physicochemical properties, suggesting good absorption. Additionally, using DataWarrior v.5.5.0 [38], we determined the toxicoinformatic properties of the compounds and eliminated those that could be tumorigenic, mutagenic, carcinogenic, or irritating: toxicoinformatic analysis to assign mutagenicity, based on the ability of a compound to cause changes in DNA through a structure comparison with molecules reported as hazardous; tumorigenicity, based on the potential of a compound to cause tumours or cancer through a structure comparison with molecules reported as hazardous; irritant based on the likelihood of a compound irritating skin, through a structure comparison with molecules reported as dangerous; and reproductive effective based on the potential for a compound to harm reproductive organs or cause developmental defects [38].

### 4.5. Development of a Web Tool and Chemical Classification

The dataset of new senotherapeutic compounds obtained from the consensus of the three algorithms was organised using a web tool based on the freely available R language package ShinyApp v1.11.1. Finally, we analysed the chemical classification to which each compound belonged to identify the chemical families.

### 4.6. Docking Analysis

We explore the potential senolytic effect of flavonoids through docking analysis between these compounds and their reported targets, as described below. It is essential to mention that proteins were selected since they have been previously reported to interact with flavonoids.

#### 4.6.1. Protein Structures Acquisition

We retrieve protein structures from the Protein Databank in the PDB format (.pdb) (https://www.rcsb.org/, accessed on 30 June 2025). The proteins retrieved are as follows: p53 (ID: 2OCJ), c-Fos (ID: 1FOS), Cyclin D1 (ID: 2W9Z), Trx (ID: 1ERT), p21 (ID: 6P8H), AKT1 (ID: 3O96), CDK1 (ID: 5HQ0), NORE1 (ID: 3DDC), p65 (ID: 6QHL), c-Jun (ID: 5T01), and p38α (ID: 3HVC).

Subsequently, protein pre-processing was generated with USCF Chimera v1.15 software [45] according to the following parameters: water residues were removed, solvent residues were removed, ions that are free or not part of the proteins were removed, coupled nucleic acids and associated ligands were removed. All protein complexes were removed, and only monomers were used for further processes.

Additionally, a protein optimisation process was generated using the USCF Chimera v1.15 interface, according to the following parameters: (1) Correction of chains and addition of hydrogens, to reflect a realistic structure and to establish the correct interactions with the ligand; (2) Partial charges, AMBER ff14SB partial charges improve the accuracy of docking, ensuring that the protein has a proper electrostatic representation, as they describe the geometry, bonds, and partial charges of the amino acids to model their interactions correctly; and (3) Energy minimisation, to reduce possible distortions, thereby improving the accuracy of the docking.

#### 4.6.2. Compounds Structure Obtention for Docking Analysis

We retrieved the 2D compound structure in .sdf format from the PubChem database (https://pubchem.ncbi.nlm.nih.gov/, accessed on 7 July 2025): 3′,4′,7-trihydroxyisoflavone (ID: 5284648), daidzin (ID: 107971), catechin (ID: 9064), eriodictyol (ID: 440735), auriculasin (ID: 5358846), pomiferin (ID: 4871), 4′-O-methylalpinumisoflavone (ID: 15596285), tephrosin (ID: 114909), 5,7,3′-trihydroxy-3,4′-dimethoxyflavone (ID: 5380905), calycosin-7-O-beta-D-glucoside (ID: 5318267), 5,7-dihydroxy-3-(3-hydroxy-4-methoxybenzyl)-6-methoxychroman-4-one (ID: 21676260), glycitein (ID: 5317750), glycitin (ID: 187808), jaceosidin (ID: 5379096), silybin (ID: 31553), isosilybin A (ID: 11059920), eupafolin (ID: 5317284), and skullcapflavone II (ID: 124211).

The 2D structures of the compounds were downloaded in .sdf format from the PubChem database and converted to three-dimensional structures using geometric optimisation performed in Avogadro v1.2.0 (https://avogadro.cc/releases/avogadro_120/) accessed on 9 December 2024, using the MMFF94 molecular mechanics algorithm. They were then prepared in UCSF Chimera v1.15 by adding hydrogens, assigning partial charges AMBER ff14SB, and minimising energy (1000 steepest descent steps and 100 conjugate gradient steps, 0.02 Å step size). The AM1-BCC force fields were applied for proteins and Gasteiger for compounds, under vacuum conditions and with the removal of non-biological residues, generating stable structures for docking analysis.

#### 4.6.3. Molecular Docking

Once the above compounds were chosen, we used the USCF Chimera version 1.15 interface to run Autodock Vina version 1.1.2 [46], a software that predicts binding modes between different target proteins and ligands.

Each compound was loaded with each protein in the USCF Chimera software, the total protein area was set, and blind docking was performed for each ligand-protein.

## 5. Conclusions

The present study identified 270 compounds with senotherapeutic potential, with flavonoids being the most abundant compound class. Notably, 18 of these compounds represent novel senolytics that warrant experimental validation due to their interesting pharmacological properties. Among these, tephrosin, 5,7-dihydroxy-3-(3-hydroxy-4-methoxybenzyl)-6-methoxychroman-4-one, and isosilybin are particularly deserving of experimental verification for further development. Nevertheless, these results are exploratory and based solely on computational models; further experimental studies and chemical modifications are necessary to improve their specificity, safety, and efficacy as senotherapeutics.

## Figures and Tables

**Table 1:** Results from three ML classifier models. Accuracy (performance), specificity (actual negative cases), recall (sensitivity), F1 (precision), and Kappa (agreement) proportions from 0 to 1.

	RF	SVM	KNN
Accuracy	0.88 *	0.76	0.76
Specificity	0.92	0.71	0.88
Precision	0.90	0.71	0.88
Recall	0.92	0.83	0.67
F1-Score	0.89	0.76	0.76
Kappa	0.76 *	0.54	0.53

* Best performance.

**Table 2:** Docking results for tested flavonoids (Table 2 and Supplementary Materials) and their protein targets according to network pharmacology (Figure 1).

Protein Target	Flavonoid	ΔG (kcal/mol)
p53	3′,4′,7-trihydroxyisoflavone	−6.3
	Catechin	−6.8
	Auriculasin	−7.4
	Glycitein	−6.1
	Silybin	−7.3
AKT1	Tephrosin	−11.2
	5,7-dihydroxy-3-(3-hydroxy-4-methoxybenzyl)-6-methoxychroman-4-one	−9.2
Trx	Pomiferin	−6.9
	Calycosin-7-O-beta-D-glucoside	−6.1
Cyclin D1	Eriodictyol	−7.2
p21	4′-O-methylalpinumisoflavone	−6.4
c-Fos	Daidzin	−6.0
CDK1	5,7,3′-trihydroxy-3,4′-dimethoxyflavone	−8.9
NORE1	Glycitin	−7.0
p65	Jaceosidin	−4.4
CDK1	Isosilybin A	−9.7
c-Jun	Eupafolin	−5.9
p38α	Skullcapflavone II	−8.5

## Data Availability

The code and models developed in this study are available at https://github.com/BioAgeLab/Senotherapeutics_Project_INGER-UACM. The database is freely available at: https://gcoixc-laboratorio0de0bioinform0tic-inger.shinyapps.io/Senotherapeutics_Shiny/.

## Supplementary Materials

The following supporting information can be downloaded at: https://www.mdpi.com/article/10.3390/ph18081176/s1, Table S1: Docking test for compounds.

## Abbreviations

The following abbreviations are used in this manuscript:

SASP	Senescence-associated secretory phenotype
AI	Artificial intelligence
ML	Machine learning
RFC	Random Forest Classifier
SVM	Supand Vector Machine
KNN	K-Nearest Neighbors
CTD	Comparative Toxicogenomics Database
PPI	Protein-Protein Interactions
ROC	Receiver Operating Characteristic
AUC	Area under the curve
FDA	Food and Drug Administration

## References

[1] Huang W., Hickson L.J., Eirin A., Kirkland J.L., Lerman L.O. (2022). Cellular Senescence: The Good, the Bad and the Unknown. Nat. Rev. Nephrol..

[2] Childs B.G., Durik M., Baker D.J., van Deursen J.M. (2015). Cellular Senescence in Aging and Age-Related Disease: From Mechanisms to Therapy. Nat. Med..

[3] Mylonas A., O’Loghlen A. (2022). Cellular Senescence and Ageing: Mechanisms and Interventions. Front. Aging.

[4] Zarneshan S.N., Fakhri S., Bachtel G., Bishayee A. (2023). Exploiting Pivotal Mechanisms behind the Senescence-like Cell Cycle Arrest in Cancer. Adv. Protein Chem. Struct. Biol..

[5] Borghesan M., Hoogaars W.M.H., Varela-Eirin M., Talma N., Demaria M. (2020). A Senescence-Centric View of Aging: Implications for Longevity and Disease. Trends Cell Biol..

[6] Lewis E.D., Wu D., Meydani S.N. (2022). Age-Associated Alterations in Immune Function and Inflammation. Prog. Neuropsychopharmacol. Biol. Psychiatry.

[7] Hazeldine J., Lord J.M. (2022). Immunesenescence and Compromised Removal of Senescent Cells: Implications for Health in Old Age. Healthy Ageing and Longevity.

[8] McHugh D., Gil J. (2018). Senescence and Aging: Causes, Consequences, and Therapeutic Avenues. J. Cell Biol..

[9] Barrera-Vázquez O.S., Magos-Guerrero G.A., Escobar-Ramírez J.L., Gomez-Verjan J.C. (2023). Natural Products as a Major Source of Candidates for Potential Senolytic Compounds Obtained by Screening. Med. Chem..

[10] Niedernhofer L.J., Robbins P.D. (2018). Senotherapeutics for Healthy Ageing. Nat. Rev. Drug Discov..

[11] Zhang L.J., Salekeen R., Soto-Palma C., He Y., Elsallabi O., Hughes B., Nunes A., Xu W., Zhang B., Mohamed A. (2024). Development of Novel Flavonoid Senolytics through Phenotypic Drug Screening and Drug Design. bioRxiv.

[12] Pacifico F., Magni F., Leonardi A., Crescenzi E. (2024). Therapy-Induced Senescence: Novel Approaches for Markers Identification. Int. J. Mol. Sci..

[13] Chen M., Fu Y., Wang X., Wu R., Su D., Zhou N., Qi Y. (2022). Metformin Protects Lens Epithelial Cells against Senescence in a Naturally Aged Mouse Model. Cell Death Discov..

[14] Walaszczyk A., Dookun E., Redgrave R., Tual-Chalot S., Victorelli S., Spyridopoulos I., Owens A., Arthur H.M., Passos J.F., Richardson G.D. (2019). Pharmacological Clearance of Senescent Cells Improves Survival and Recovery in Aged Mice Following Acute Myocardial Infarction. Aging Cell.

[15] Islam M.T., Tuday E., Allen S., Kim J., Trott D.W., Holland W.L., Donato A.J., Lesniewski L.A. (2023). Senolytic Drugs, Dasatinib and Quercetin, Attenuate Adipose Tissue Inflammation, and Ameliorate Metabolic Function in Old Age. Aging Cell.

[16] Hickson L.J., Langhi Prata L.G.P., Bobart S.A., Evans T.K., Giorgadze N., Hashmi S.K., Herrmann S.M., Jensen M.D., Jia Q., Jordan K.L. (2019). Senolytics Decrease Senescent Cells in Humans: Preliminary Report from a Clinical Trial of Dasatinib plus Quercetin in Individuals with Diabetic Kidney Disease. EBioMedicine.

[17] Alzforum Dasatinib + Quercetin. https://www.alzforum.org/therapeutics/dasatinib-quercetin.

[18] He Y., Zheng G., Zhou D. (2020). Senolytic Drug Development. Healthy Ageing and Longevity.

[19] Muthuramalingam P., Jeyasri R., Varadharajan V., Priya A., Dhanapal A.R., Shin H., Thiruvengadam M., Ramesh M., Krishnan M., Omosimua R.O. (2024). Network Pharmacology: An Efficient but Underutilized Approach in Oral, Head and Neck Cancer Therapy-a Review. Front. Pharmacol..

[20] Smer-Barreto V., Quintanilla A., Elliott R.J.R., Dawson J.C., Sun J., Campa V.M., Lorente-Macías Á., Unciti-Broceta A., Carragher N.O., Acosta J.C. (2023). Discovery of Senolytics Using Machine Learning. Nat. Commun..

[21] Meenakshi M., Ruban S., Nandhini T.J., Loganayagi S., Sayed R.M., Hassan E., Al-Saidi I.A.-D.H. A Framework Design of ML Classifier Algorithm for Retrieve the Information about the Drugs and Its Quality. Proceedings of the 2024 4th International Conference on Advance Computing and Innovative Technologies in Engineering (ICACITE).

[22] Priya S., Tripathi G., Singh D.B., Jain P., Kumar A. (2022). Machine Learning Approaches and Their Applications in Drug Discovery and Design. Chem. Biol. Drug Des..

[23] Ferreira F.J.N., Carneiro A.S. (2025). AI-Driven Drug Discovery: A Comprehensive Review. ACS Omega.

[24] Shah A., Patel V., Jain M., Parmar G. (2023). Network Pharmacology and Systems Biology in Drug Discovery. Interdisciplinary Biotechnological Advances.

[25] Arrell D.K., Terzic A. (2010). Network Systems Biology for Drug Discovery. Clin. Pharmacol. Ther..

[26] Knegtel R.M.A., Grootenhuis P.D.J. (2005). Binding Affinities and Non-Bonded Interaction Energies. 3D QSAR in Drug Design.

[27] Kong X., Xu J., Hussain S.A., Alrubie T.M., Maddu N., Wei H. (2025). Combination of Quercetin and Curcumin modulates NF-κB inflammatory signaling pathway in murine model of esophageal erosive reflux disease erosive reflux disease. Folia Morphol. (Warsz).

[28] Ahmadinejad F., Bos T., Hu B., Britt E., Koblinski J., Souers A.J., Leverson J.D., Faber A.C., Gewirtz D.A., Harada H. (2022). Senolytic-Mediated Elimination of Head and Neck Tumor Cells Induced Into Senescence by Cisplatin. Mol. Pharmacol..

[29] Proshkina E., Koval L., Platonova E., Golubev D., Ulyasheva N., Babak T., Shaposhnikov M., Moskalev A. (2024). Polyphenols as Potential Geroprotectors. Antioxid. Redox Signal..

[30] Tiwari A.K., Singh M.V. (2023). Insights into the Origin and Therapeutic Implications of Benzopyran and Its Derivatives. ChemistrySelect.

[31] Yuan D., Guo Y., Pu F., Yang C., Xiao X., Du H., He J., Lu S. (2024). Opportunities and Challenges in Enhancing the Bioavailability and Bioactivity of Dietary Flavonoids: A Novel Delivery System Perspective. Food Chem..

[32] Zhao J., Yang J., Xie Y. (2019). Improvement Strategies for the Oral Bioavailability of Poorly Water-Soluble Flavonoids: An Overview. Int. J. Pharm..

[33] Vallance T.M., Ravishankar D., Albadawi D.A.I., Osborn H.M.I., Vaiyapuri S. (2019). Synthetic Flavonoids as Novel Modulators of Platelet Function and Thrombosis. Int. J. Mol. Sci..

[34] Bisson J., McAlpine J.B., Friesen J.B., Chen S.-N., Graham J., Pauli G.F. (2016). Can Invalid Bioactives Undermine Natural Product-Based Drug Discovery?. J. Med. Chem..

[35] Baell J., Walters M.A. (2014). Chemistry: Chemical Con Artists Foil Drug Discovery. Nature.

[36] Uivarosi V., Munteanu A.-C., Nițulescu G.M. (2019). An Overview of Synthetic and Semisynthetic Flavonoid Derivatives and Analogues: Perspectives in Drug Discovery. Studies in Natural Products Chemistry.

[37] Mattingly C.J., Rosenstein M.C., Davis A.P., Colby G.T., Forrest J.N., Boyer J.L. (2006). The Comparative Toxicogenomics Database: A Cross-Species Resource for Building Chemical-Gene Interaction Networks. Toxicol. Sci..

[38] Sander T., Freyss J., von Korff M., Rufener C. (2015). DataWarrior: An Open-Source Program for Chemistry Aware Data Visualization and Analysis. J. Chem. Inf. Model..

[39] Salman H.A., Kalakech A., Steiti A. (2024). Random Forest Algorithm Overview. Babylon. J. Mach. Learn..

[40] Schonlau M. (2023). Support Vector Machines. Statistics and Computing.

[41] Mladenova T., Valova I. (2023). Classification with K-Nearest Neighbors Algorithm: Comparative Analysis between the Manual and Automatic Methods for K-Selection. Int. J. Adv. Comput. Sci. Appl..

[42] Zhang L., Pitcher L.E., Prahalad V., Niedernhofer L.J., Robbins P.D. (2023). Targeting Cellular Senescence with Senotherapeutics: Senolytics and Senomorphics. FEBS J..

[43] Naidu G., Zuva T., Sibanda E.M. (2023). A Review of Evaluation Metrics in Machine Learning Algorithms. Lecture Notes in Networks and Systems.

[44] Pillai O., Dhanikula A.B., Panchagnula R. (2001). Drug Delivery: An Odyssey of 100 Years. Curr. Opin. Chem. Biol..

[45] Pettersen E.F., Goddard T.D., Huang C.C., Couch G.S., Greenblatt D.M., Meng E.C., Ferrin T.E. (2004). UCSF Chimera--a Visualization System for Exploratory Research and Analysis. J. Comput. Chem..

[46] Trott O., Olson A.J. (2010). AutoDock Vina: Improving the Speed and Accuracy of Docking with a New Scoring Function, Efficient Optimization, and Multithreading. J. Comput. Chem..

