# Supplementary material for: Network Pharmacology and Machine Learning Identify Flavonoids as Potential Senotherapeutics
Source: Pharmaceuticals (Basel). 2025 Aug 9;18(8):1176. doi: 10.3390/ph18081176 (PMC12389116; doi:10.3390/ph18081176)
Supplement: Supplementary file 1 [file pharmaceuticals-18-01176-s001.zip › pharmaceuticals-3729208-supplementary.pdf]

Table S1. Docking test for compounds.

Ligand Flavonoid	Protein	ΔG (kcal/mol)	Docking
3',4',7-trihydroxyisoflavone	p53	-6.3	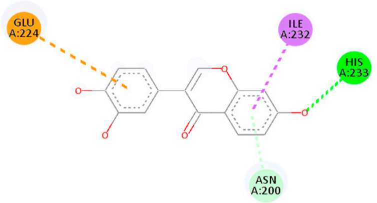
Daidzin	c-Fos	-6.0	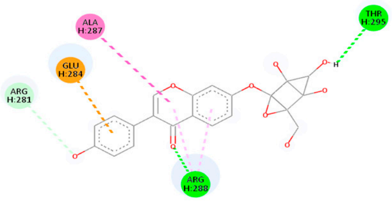
Catechin	p53	-6.8	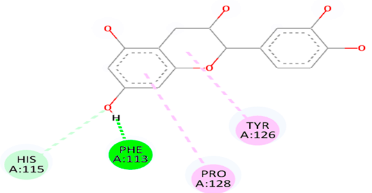
Eriodictyol	Cyclin D1	-7.2	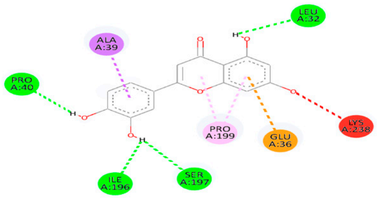
Auriculasin	p53	-7.4	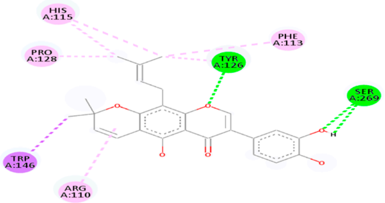
Pomiferin	Trx	-6.9	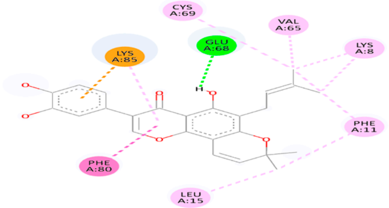

4'-O-methylalpinumisoflavone	p21	-6.4	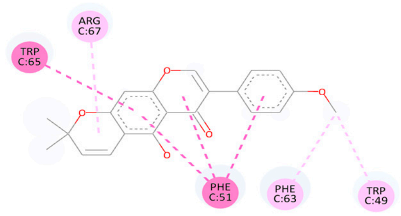
Tephrosin	AKT1	-11.2	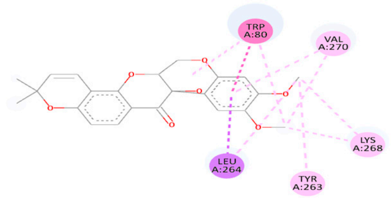
5,7,3'-trihydroxy-3,4'-dimethoxyflavone	CDK1	-8.9	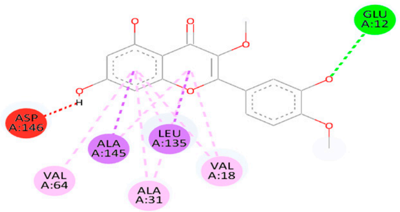
Calycosin-7-O-beta-D-glucoside	Trx	-6.1	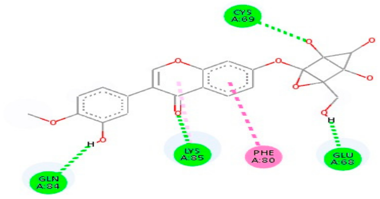
5,7-dihydroxy-3-(3-hydroxy-4-methoxybenzyl)-6-methoxychroman-4-one	AKT1	-9.2	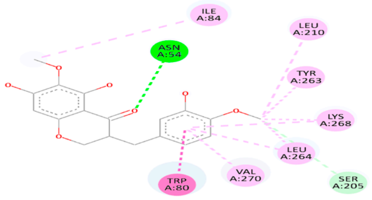
Glycitein	p53	-6.1	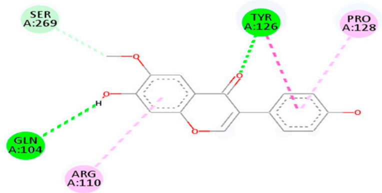
Glycitin	NORE1	-7.0	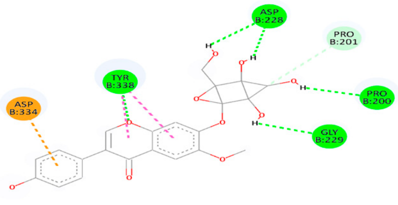

Jaceosidin	p65	-4.4	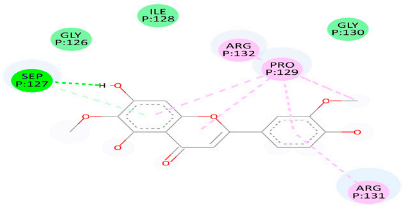
Silybin	p53	-7.3	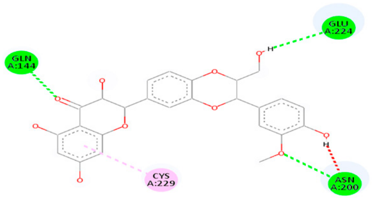
Isosilybin A	CDK1	-9.7	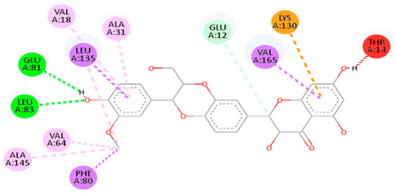
Eupafolin	c-Jun	-5.9	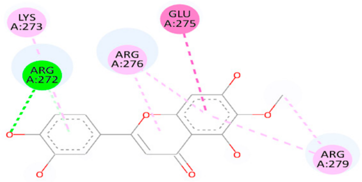
Skullcapflavone II	p38α	-8.5	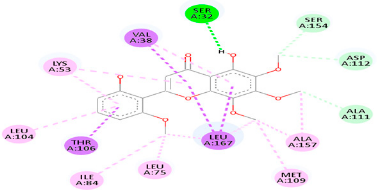

Protein: p53<br>Ligand: 3',4',7-trihydroxyisoflavone<br>ΔG: -6.3<br>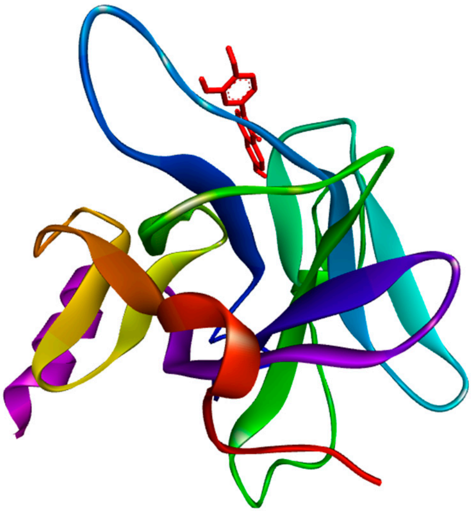<br>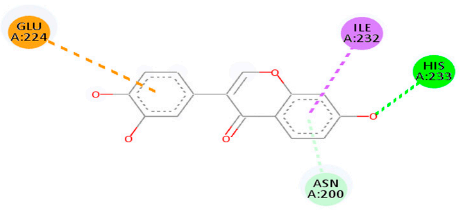 	Protein: c-Fos<br>Ligand: Daidzin<br>ΔG: -6.0<br>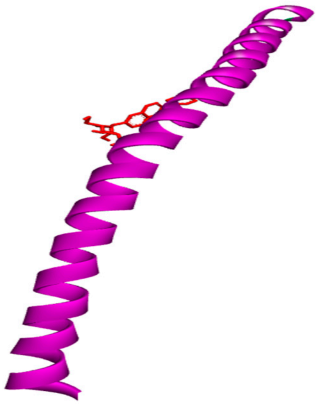<br>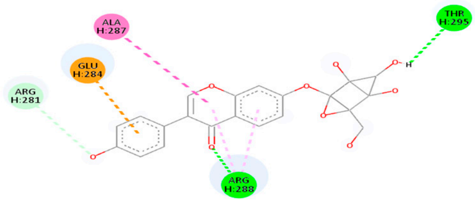
Protein: p53<br>Ligand: Catechin	Protein: CiclinD<br>Ligand: Eriodictyol

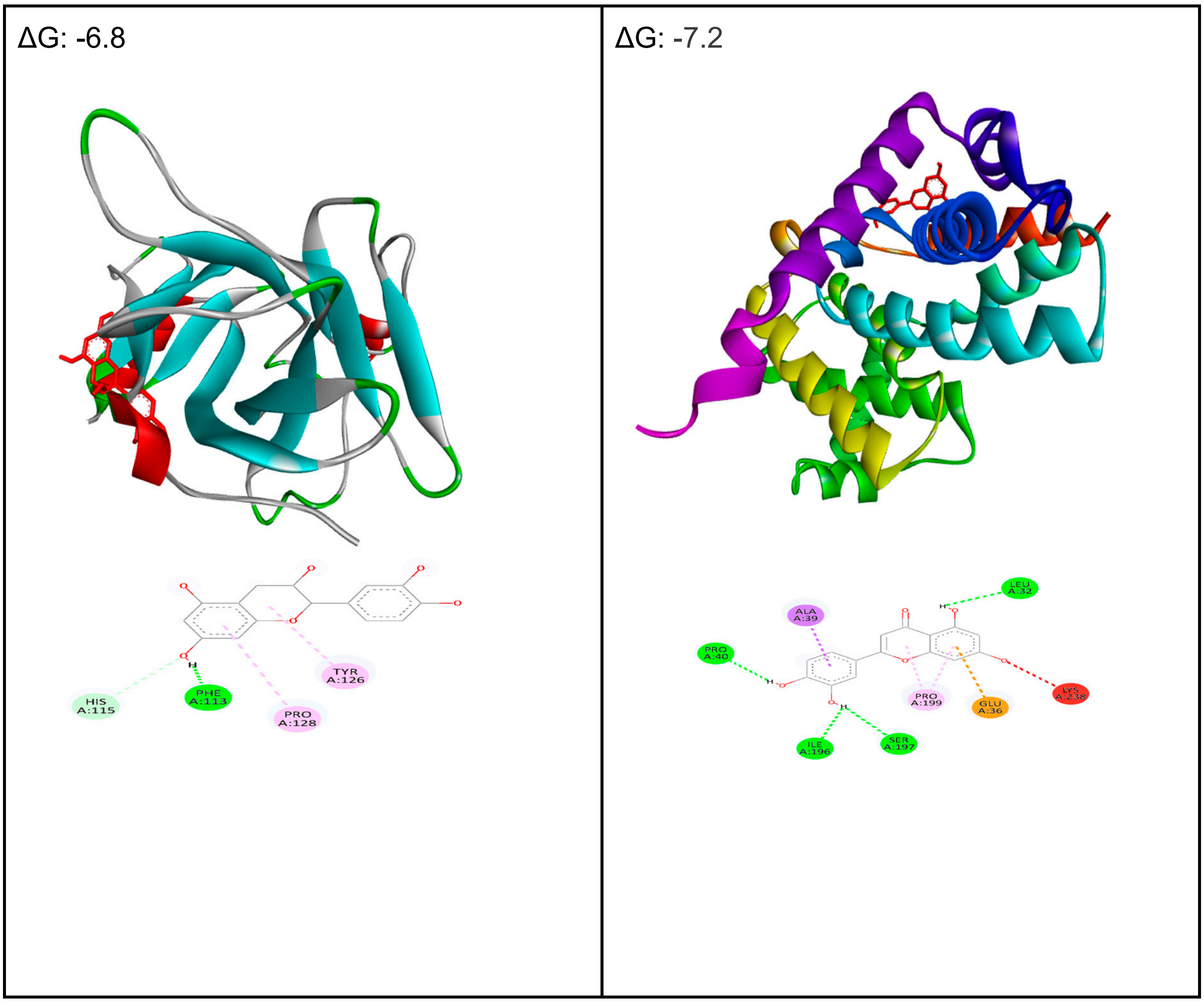
ΔG: -6.8
HIS A:115
PHE A:113
TYR A:126
PRO A:128
ΔG: -7.2
LEU A:32
ALA A:39
PRO A:40
PRO A:199
GLU A:36
LYS A:238
ILE A:196
SER A:197

<table>
<tr>
<td>
Protein: p53
Ligand: Auriculasin
ΔG: -7.4
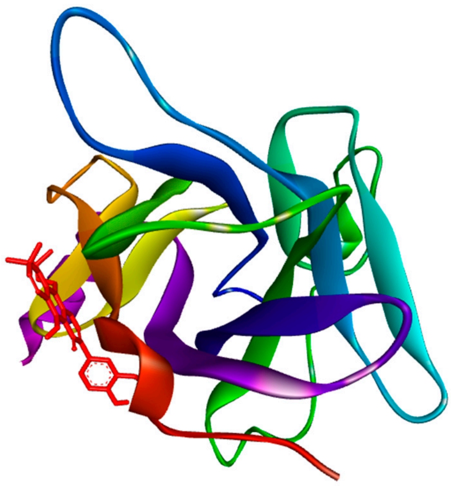
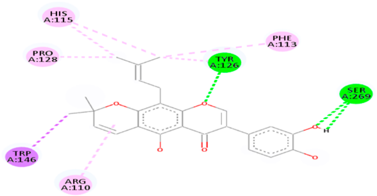

</td>
<td>
Protein: Trx
Ligand: Pomiferin
ΔG: -6.9
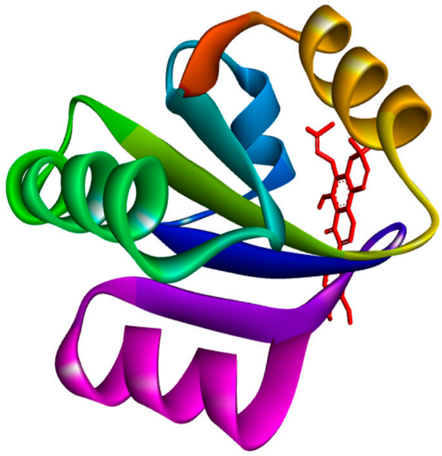
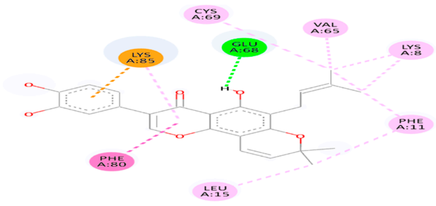

</td>
</tr>
</table>

Protein: p21
Ligand: 4'-O-methylalpinumisoflavone
ΔG: -6.4

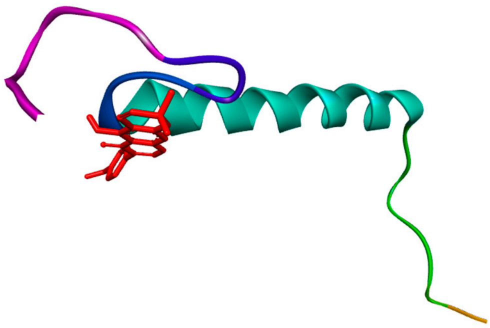

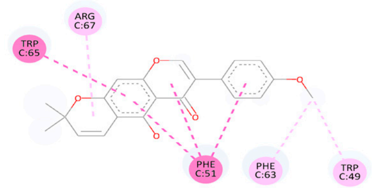

Protein: AKT
Ligand: Tephrosin
ΔG: -11.2

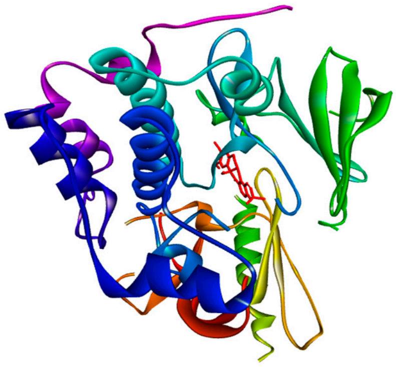

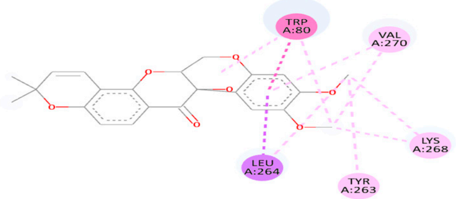

Protein: CDK1
Ligand: 5,7,3'-trihydroxy-3,4'-dimethoxy flavone
ΔG: -8.9

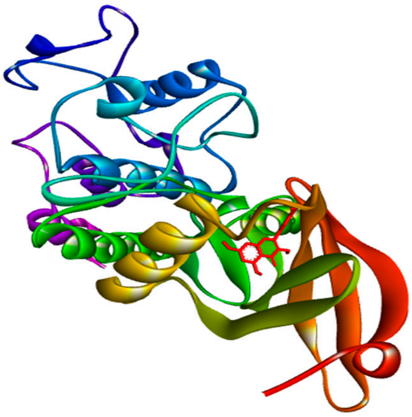

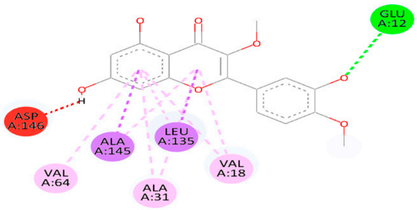

Protein: Trx
Ligand: Calycosin-7-O-beta-D-glucoside
ΔG: -6.1

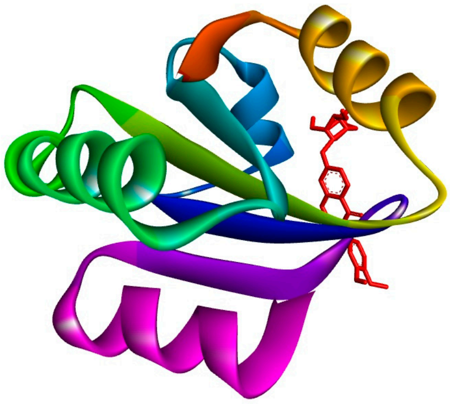

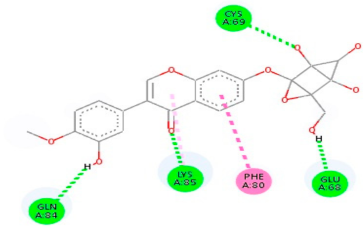

Protein: AKT
Ligand: 5,7-dihydroxy-3-(3-hydroxy-4-methoxybenzyl)-6-methoxychroman-4-one
ΔG: -9.2

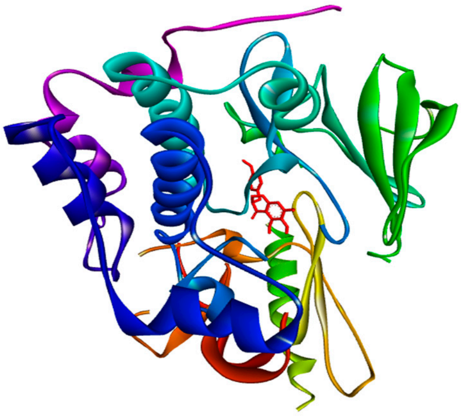

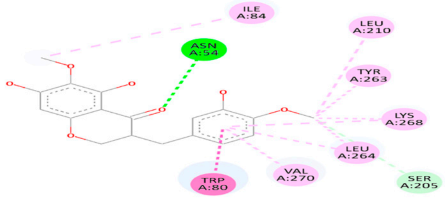

Protein: p53
Ligand: Glycitein
ΔG: -6.1

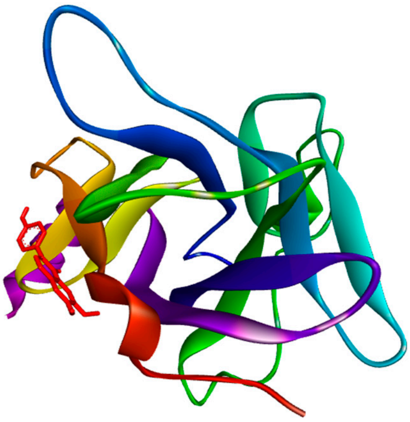

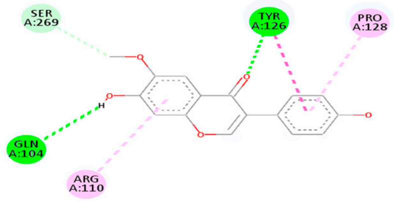

Protein: NORE 1
Ligand: Glycitin
ΔG: -7.0

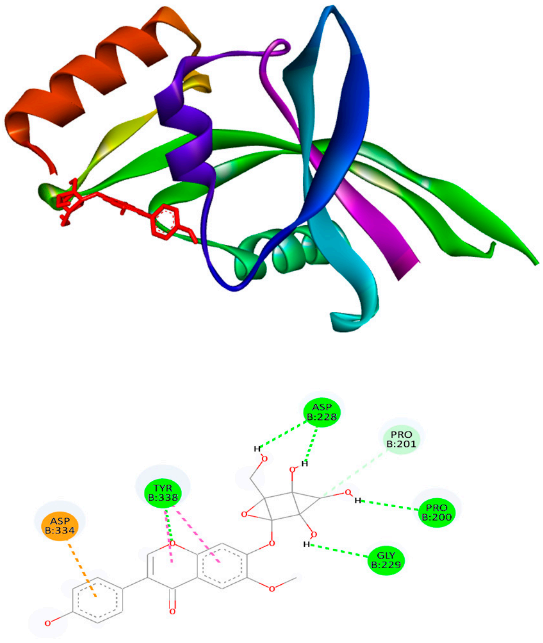

Protein: p65
Ligand: Jaceosidin
ΔG: -4.4

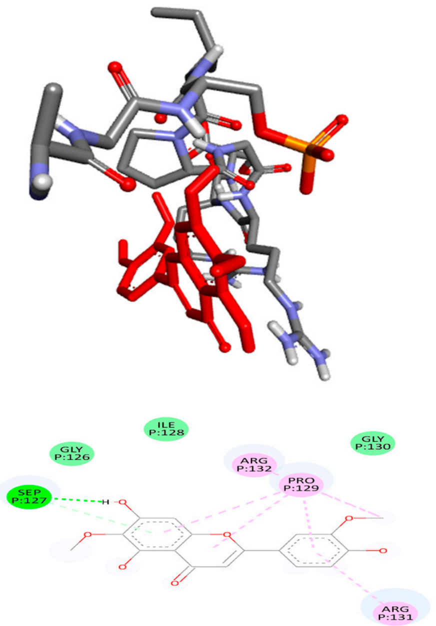

Protein: p53
Ligand: Silybin
ΔG: -7.3

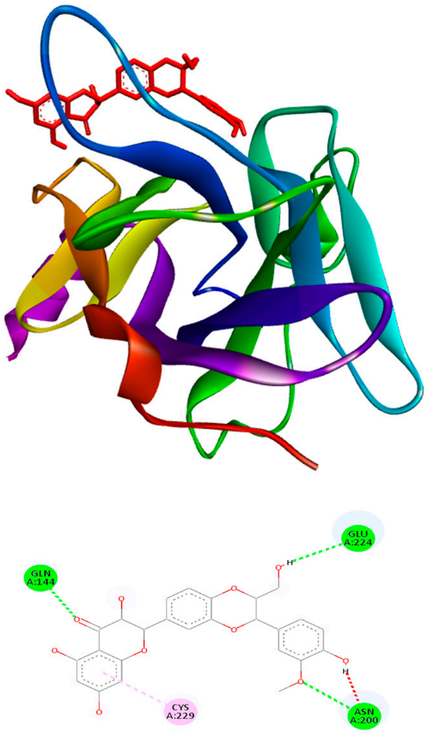

Protein: CDK1
Ligand: Isosilybin A
ΔG: -9.7

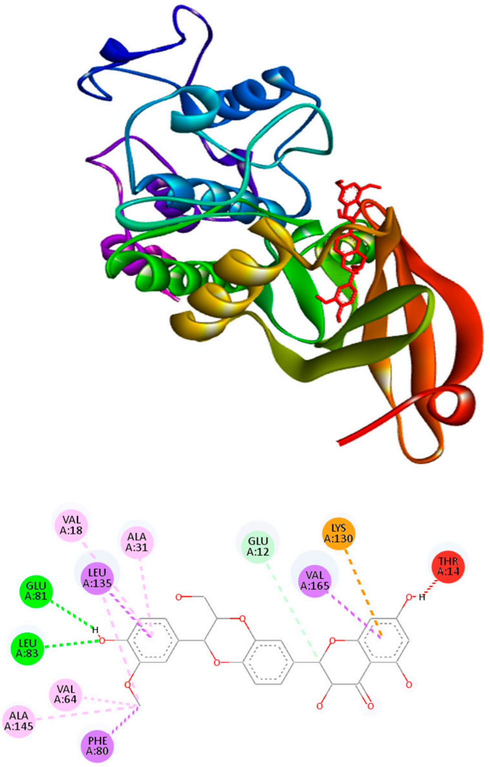

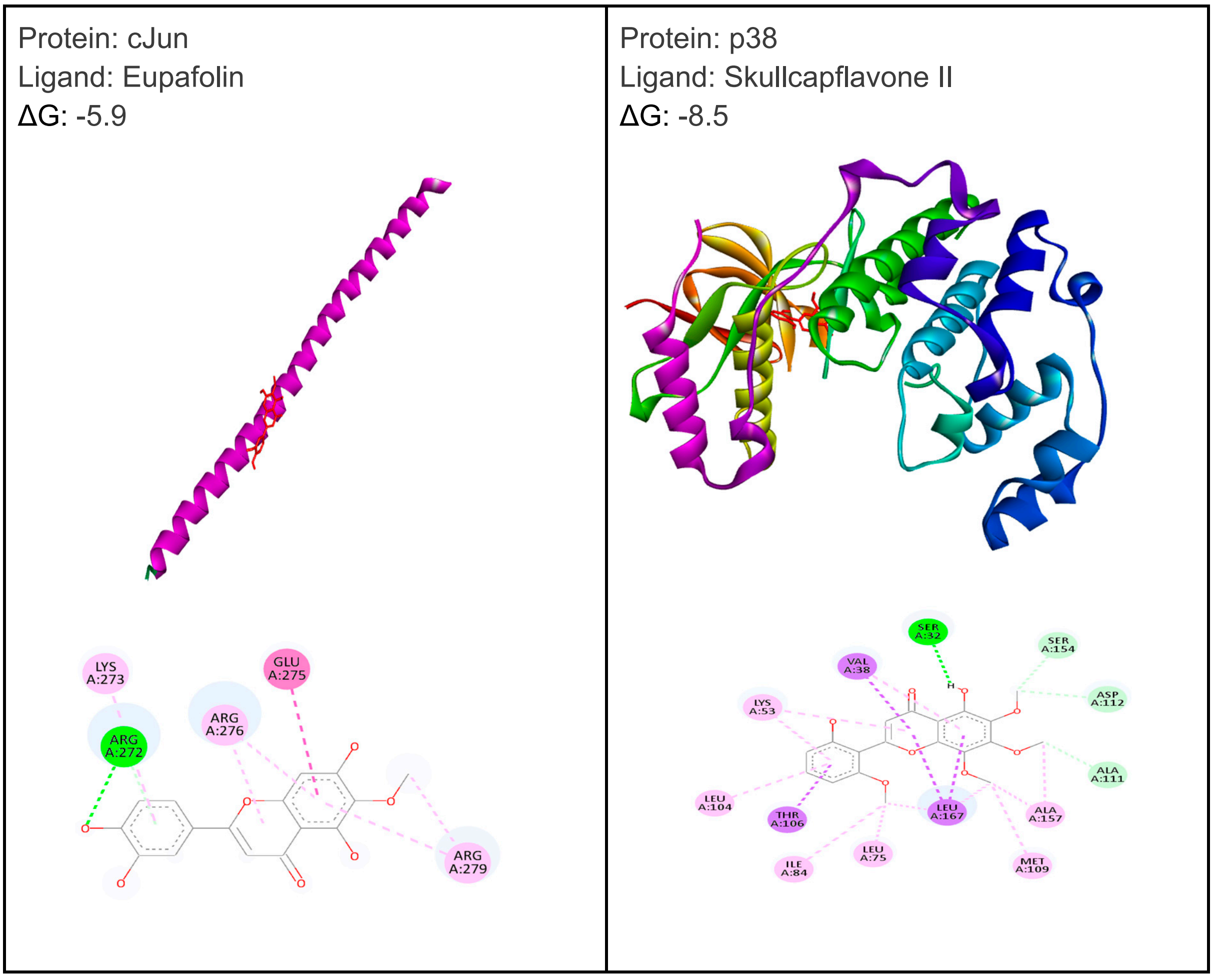
Protein: cJun
Ligand: Eupafolin
ΔG: -5.9
LYS A:273
GLU A:275
ARG A:272
ARG A:276
ARG A:279
Protein: p38
Ligand: Skullcapflavone II
ΔG: -8.5
SER A:32
SER A:154
VAL A:38
LYS A:53
ASP A:112
ALA A:111
LEU A:104
THR A:106
LEU A:167
ALA A:157
LEU A:75
ILE A:84
MET A:109
